# Discordant biochemical parameters of acromegaly remission do not influence the prevalence or aggressiveness of metabolic comorbidities: a single-center study

**DOI:** 10.3389/fendo.2023.1256975

**Published:** 2023-09-27

**Authors:** Martina Romanisio, Rosa Pitino, Alice Ferrero, Francesca Pizzolitto, Samuele Costelli, Valentina Antoniotti, Paolo Marzullo, Gianluca Aimaretti, Flavia Prodam, Marina Caputo

**Affiliations:** ^1^ Endocrinology, Department of Translational Medicine, Università del Piemonte Orientale, Novara, Italy; ^2^ Department of Health Sciences, Università del Piemonte Orientale, Novara, Italy

**Keywords:** Acromegaly, IGF-1, GH, discrepancy, metabolic, complications

## Abstract

**Purpose:**

The discrepancy between the biomarkers of disease’s activity in acromegalic patients (GH and IGF-1) is almost frequent representing a challenge for the development of comorbidities in the long term. The aim of this study was to evaluate the prevalence and severity of metabolic comorbidities (diabetes, hypertension, and dyslipidemia) in surgically treated acromegalic patients with disease control and discordant GH and/or IGF-1 levels compared with those with concordant values.

**Patients and methods:**

Retrospective monocentric observational study on acromegalic surgically treated patients with biochemical remission (group A) or mild discordant GH or IGF-1 levels (group B). Metabolic complications and medical therapy were assessed at diagnosis and at the last follow-up visit. Severity of the disease was set for drug titration or shift to another molecule or more than before.

**Results:**

There were 18 patients that met the inclusion criteria [group A: nine patients; group B: nine patients, follow-up 7 years (IQR 5.0;11.25)]. The prevalence of female patients was significantly higher in the remission group compared with the discordant group (p < 0.02). Considering metabolic complications, at the last follow-up, 61.1% was affected by hypertension, 33.3% by diabetes, and 61.1% by dyslipidemia, without differences between groups. Drug characteristics (dose, shift, number) during the follow-up did not differ significantly between groups.

**Conclusion:**

Metabolic complications, mainly dyslipidemia, are frequent in cured acromegalic patients, but GH/IGF-1 discrepancy does not seem to represent a risk factor for their presence or persistence. More extended studies are needed to confirm our results in a long-term period.

## Introduction

Acromegaly is a rare systemic pathology resulting from a growth hormone (GH)-secreting pituitary adenoma (1). The worldwide estimated prevalence is 40–130 per million inhabitants and incidence of 3–5 per million person years (2), but recent studies showed higher rates than previously reported (3). Elevated GH levels lead to liver hyperproduction of insulin-like growth factor 1 (IGF-1), causing somatic modifications and systemic manifestations [i.e., cardiovascular disease, osteoarthropathy, metabolic complications, obstructive sleep apnea (OSAS), hyperhidrosis, carpal tunnel syndrome] (4). Nevertheless, the diagnosis is usually 5 to 10 years delayed; thus, complications of the GH/IGF-I excess are frequent (5).

Active disease is still associated with increased mortality, although recent studies have demonstrated that the disease control could reduce the mortality risk as for general population thanks to novel treatments (6). Cardiovascular diseases have been counted as the primary cause of death for many years (7). In fact, acromegaly is characterized by high prevalence of risk factors for coronary heart disease such as arterial hypertension, hyperglycemia, and dyslipidemia. Up to 60% of acromegalic patients are affected by hypertension (8), caused by different effects (i.e., GH/IGF-1 are anti-natriuretic, the enhancement of the peripheral vascular resistance, and the onset of sleep apnea syndrome) leading to extracellular fluid volume expansion (9). In up to 50% of patients, impaired glucose tolerance and secondary diabetes occur (10), since GH hyperproduction leads to insulin resistance; thus, a particular cardiomyopathy could be detected in acromegaly complicated by hyperglycemia. Furthermore, dyslipidemia is frequent, related to different factors such as the release of free fatty acids (FFAs) on bloodstream from the liver, and the occurrence of insulin resistance; it is characterized by increase of triglycerides and decrease of high-density lipoprotein (HDL) levels (11). Thus, acromegalic patients are characterized by a higher Framingham risk score than normal subjects, caused by high blood pressure, dysglycemia, and hyperlipidemia (12).To define the biochemical control of acromegaly, the Endocrine Society suggests the goal of a random GH less than 1 μg/L and normal IGF-1 for age and sex (2). In the majority of patients, GH and IGF-1 levels are concordant, pointing out remission or active disease; however, in up to 25% of acromegalic patients who underwent surgery, incongruent GH and IGF-1 levels have been described (13). The effect of the incongruence of GH and IGF-1 values on acromegaly comorbidities has been studied with discordant results on the onset or progression of the metabolic complications (14–16).

Based on the above, this retrospective monocentric observational study aimed to estimate the prevalence and severity of metabolic comorbidities (diabetes, hypertension, and dyslipidemia) in surgically treated acromegalic patients with disease control and concordant or discordant GH/IGF-1 levels. The choice to describe a single-center experience ensures a standardized management.

## Patients and methods

### Patients

An observational, retrospective, single-center study was performed. Patients affected by acromegaly referring to the Neuroendocrinology Unit of “Maggiore della Carità” University Hospital in Novara between 01/01/2007 and 31/03/2022 were consecutively recruited.

Clinical, hormonal, and radiologic characteristics of all subjects were evaluated through the review of endocrine clinical records.

For each patient, the following data were collected: demographic features (gender, age at diagnosis, and age at surgery); magnetic resonance imaging (MRI) radiological parameters at diagnosis (micro- vs. macroadenoma, maximum diameter) and during follow-up; presence of mass effect (i.e., alteration of visual field); biochemical and hormonal evaluation at diagnosis and during follow-up (random GH and/or GH nadir during 75 g oral glucose tolerance test (OGTT), and IGF-1 levels); histopathological characteristics (immunohistochemical features, Ki67); medical treatment for acromegaly such as somatostatin analogs (SSAs: lanreotide, octreotide, pasireotide), dopamine agonists (cabergoline, bromocriptine), growth hormone receptor antagonists (GHRAs: pegvisomant), and/or radiation therapy; and presence and severity of metabolic complications (diabetes mellitus, arterial hypertension, dyslipidemia); their severity was evaluated according to titration of dose or shift to another or more drugs.

Acromegalic patients were included in the study if the following inclusion criteria were satisfied: (i) surgically treated GH-secreting pituitary adenoma, demonstrated by GH positivity at immunohistochemistry (IHC) on pathological examination; (ii) biochemical assessment of somatotroph axis (IGF-1 and random GH or GH after 75g OGTT) after neurosurgery and at last follow-up; (iii) biochemical remission or mild discordant GH or IGF-1 levels; (iv) evaluation of metabolic comorbidities.

We exclude from the study patients with confounding conditions on GH/IGF-1 secretion (i.e., pregnancy, puberty, estrogen treatment, chronic kidney disease, liver insufficiency, and untreated hypothyroidism).

According to the current guidelines, postsurgical criteria for remission were defined in case of normal IGF-1 levels for age and sex and random GH less than 1 μg/L (2), as they correlate with control of acromegaly. Hormonal discordance was defined as random GH ≥1 μg/L or nadir GH level after a glucose load ≥1 μg/L with normal IGF-1 levels for age and sex or as elevated IGF-1 levels with a random GH or GH after 75 g OGTT <1 μg/L were documented.

Thus, patients were divided into two groups considering to the biochemical status: group A consisted of patients with a biochemical remission, group B with discordant GH/IGF-1 values.

The study was conducted in accordance with the Declaration of Helsinki, approved by the Local Ethical Committee (AOU “Maggiore della Carità” Novara). Informed consent was obtained from each patient.

### Methods

#### Biochemical evaluation

GH measurement was performed by chemiluminescence GH assay (LIAISON^®^), and IGF-1 by LIAISON^®^ IGF-1 assay at the Biochemistry Laboratory of our Hospital. Samples were collected in the morning after an overnight fasting.

#### Metabolic complications

We described metabolic complications (diabetes mellitus, impaired fasting glucose (IFG), impaired glucose tolerance (IGT), arterial hypertension, dyslipidemia) at diagnosis and during follow-up.

The presence of diabetes mellitus, IFG, or IGT was assessed according to current guidelines (17) based on plasma glucose criteria, either the fasting plasma glucose value or the 2-h plasma glucose value during a 75-g OGTT, or glycated hemoglobin (A1C) criteria. Dyslipidemia was diagnosed and treated according to patients’ cardiovascular risk (18). Arterial hypertension was detected, evaluated, and managed according to the most recent guidelines (19). Number of treatments, shift to another or more drugs, and dose titration during follow-up were assessed.

#### Statistical analysis

Data were expressed as percentages or median ± interquartile range (IQR) We performed statistical comparisons of quantitative data with the non-parametric Mann–Whitney–Wilcoxon test or ANOVA (Kruskal–Wallis test) due to the sample size. For statistical comparisons of dichotomous data, we used the χ^2^ test. Spearman’s correlation analysis was also performed. All statistical tests were two sided with p values of <0.05 considered significant. All the statistical analyses were performed by using SPSS 27.0 (IBM SPSS Inc., Chicago, IL, USA).

## Results

**Figure 1:**
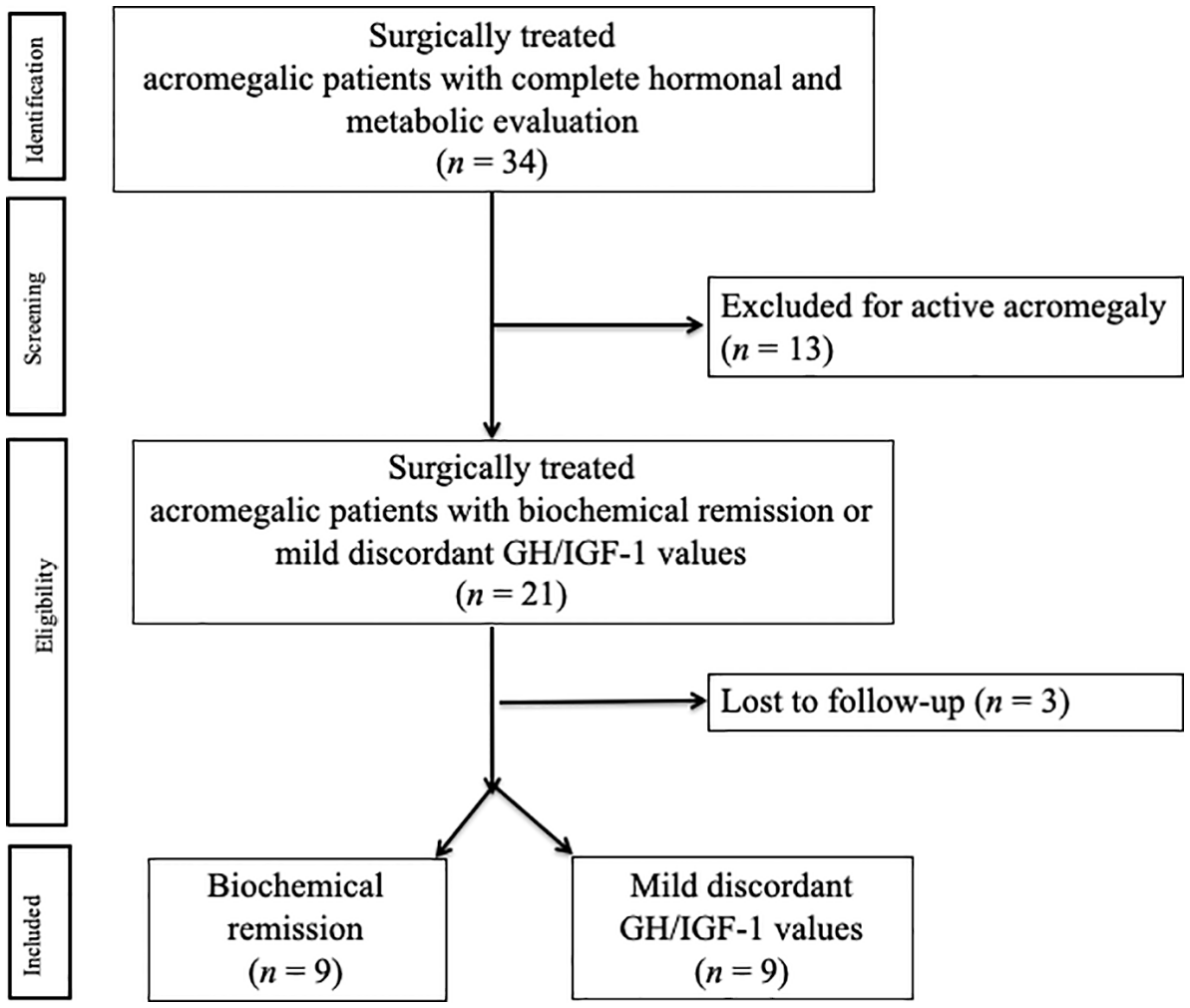
Flow diagram of patients included in the study.

There were 18 acromegalic patients enclosed in the study as they met the inclusion criteria (**Figure 1**).

According to the biochemical status during follow-up, nine patients (50%) were enclosed in group A (biochemical remission) and nine patients (50%) in group B (discordant GH/IGF-1 values). Among the discordant group (group B), six patients (66.7%) were “High IGF-1” and three (33.3%) were “High GH”.

Demographic and clinical characteristics of the subjects are summarized in **Table 1**. Considering the whole population, most of the patients were women (61.1%, n = 11). The age at diagnosis was 52.0 years (44.75;66.75). All patients were affected by a GH-secreting adenoma (83.3% macroadenomas, 26.3% microadenomas); one patient only (5.6%) presented hyperprolactinemia at diagnosis. The median diameter adenoma was 12.0 mm (10.0;18.0), and 2 out of 15 macroadenomas (13.0%) invaded the cavernous sinus.

**Table 1:** Demographic and clinical characteristics of patients at diagnosis.

	Total (n = 18)	Group A (biochemical remission) (n = 9)	Group B (discordant GH/IGF-1) (n = 9)	p value
Female, n (%)	11 (61.1)	8 (88.9)	3 (33.3)	p < 0.02
Age at diagnosis (years), median (IQR)	53 (44.75;66.75)	62 (40.5;72)	50 (46;59.5)	NS
Macroadenoma, n (%)	15 (83.3)	7 (77.8)	8 (88.9)	NS
Size (mm), median (IQR)	12 (10;18)	11 (9.5;15)	16 (11.5;21)	NS
Random GH (ng/ml), median (IQR)	7.2 (5.5;11.86)	7.2 (5.5;12.8)	6.95 (5.11;13.68)	NS
IGF-1 (ng/ml), median (IQR)	636 (470.5;884.5)	532 (421.5;798.5)	672 (536;933.25)	NS
Neurosurgery, n (%)	16 (88.9)	7 (77.8)	9 (100)	NS
Medical therapy, n (%)	13 (72.2)	6 (66.7)	7 (77.8)	NS

NS, not significant.

Median follow-up was 7 years [(5.0;11.25); group A: 9 years (4.5;11.5) group B: 6 years (5;11.5)] without significant differences between groups.

### Patients’ characteristics at diagnosis and treatment

At diagnosis, the median IGF-1 was 636.0 ng/ml (470.5;884.5). The median random GH was 7.2 ng/ml (5.5;11.865), and the median OGTT GH nadir was 3.2 ng/ml (1.6;7.36). Two patients had partial pituitary insufficiency (11.1%), and three patients had visual field impairment (16.7%).

Most of the subjects (88.9%, n = 16) underwent neurosurgical treatment, and the mean age at neurosurgery (NS) was 53.0 years (46.5;73.0); a residual adenoma was found in three cases (16.7%). Six months after NS, the median IGF-1 was 213.5 ng/ml (160.825;309.4); the median random GH was 0.19 ng/ml (0.13;1.225), and the median GH nadir after OGTT was 0.1 ng/ml (0.05;0.38).

After NS, 11 patients were in remission (68.8%) whereas 5 patients (31.2%) required adjuvant therapy (3 DA, 1 SSA, 1 DA+SSA), achieving an adequate disease control.Two patients underwent biopsy of the pituitary lesion in another center, confirming the diagnosis of pituitary adenoma with GH positivity at IHC. They refused subsequent surgical treatment and were in SSA therapy in adequate disease control.

Regarding comorbidities at diagnosis, nine patients (50%) were affected by heart disease [eight of them (88.9%) by hypertrophic cardiomyopathy and one (11.1%) by ischemic heart disease], nine patients (50%) by arterial hypertension, four patients (22.2%) by diabetes mellitus, seven patients (38.9%) by dyslipidemia, five patients (27.8%) by osteopenia/osteoporosis (one complicated by a vertebral fracture treated with bisphosphonate), 10 patients (55.6%) by benign thyroid nodules, four patients (22.2%) by colon polyposis, two patients by OSAS (11.1%), and two patients (11.1%) by carpal tunnel syndrome.

Regarding metabolic alterations, among the nine patients with arterial hypertension, 66.7% were taking a multidrug therapy (n = 6). Considering the four patients with diabetes mellitus, 50% (n = 2) were in nutritional therapy, one patient was treated by metformin and basal glargine insulin, and one patient was treated with multiple daily insulin injections. Two out of seven patients with dyslipidemia were taking statin treatments (with 30% of potency in lowering cholesterol levels).

### Patients’ characteristics at the last follow-up

At the last follow-up, the mean age was 63.5 years (51.0;77.25). Considering biochemical parameters, the median IGF-1 was 176.2 ng/ml (123.6;213.5); the median random GH was 0.91 ng/ml (0.405;1.12) (**Table 2**). IGF-1 at last follow-up was lower than values 6 months after NS (p < 0.05).

**Table 2:** Patients’ clinical characteristics at last follow-up.

	Total (n = 18)	Group A (biochemical remission) (n = 9)	Group B (discordant GH/IGF-1) (n = 9)	p value
Age (years), median (IQR)	63.5 (51;77.25)	70 (48.5;80)	62 (51;69)	NS
IGF-1 (ng/ml), median (IQR)	176.2 (123;213.5)	126.3 (105.025;166.325)	208 (169.55;296.2)	p < 0.02
Random GH (ng/ml), median (IQR)	0.91 (0.405;1.12)	0.42 (0.38;1)	1.12 (0.8825;1.61)	NS
Pituitary deficit, n (%)	2 (11.1)	1 (5.6)	1 (5.6)	NS
Visual field impairment, n (%)	0	0	0	NS

NS, not significant.

According to differences of group A vs. group B, the prevalence of female patients was significantly higher in the remission group than in the discordant group (p < 0.02, χ^2^: 5.519). Moreover, at the last follow-up, IGF-1 levels were higher in the discordant group [median IGF-1 208.0 ng/ml (169.55;296.2) vs. 126.3 (105.025;166.325), p < 0.02], as expected.

In the whole population, a negative correlation between IGF-1 values at last follow-up and age at diagnosis (r: −0.471, p = 0.05) was found; conversely, a positive correlation between IGF-1 values at last follow-up and IGF-1 at diagnosis (r: 0.559, p < 0.02) persisted. Finally, IGF-1 at last follow-up was negatively related to the duration of the therapy with SSA (r: −0.461, p = 0.06) nearly to significance.

Considering metabolic complications at last follow-up, 11 patients (61.1%) were affected by arterial hypertension, 5 (27.8%) in the remission group and 6 (33.3%) in the discordant group; 6 patients (33.3%) were affected by diabetes mellitus, 3 (16.7%) in the remission group, and 3 (16.7%) in the discordant group; 11 patients (61.1%) were affected by dyslipidemia, 5 (27.8%) in the remission group and 6 (33.3%) in the discordant group. No differences of prevalence between groups were found.

Regarding medical treatment for metabolic complications, four out of 11 patients with arterial hypertension took one drug (two in the remission group and two in the discordant group) whereas seven took more than one treatment (three in the remission group and four in the discordant group). Moreover, five patients (27.8%) should potentiate antihypertensive therapy since diagnosis (two in the remission group, three in the discordant group).

Considering the six patients affected by diabetes mellitus, four were in nutritional therapy whereas two were in medical treatment (one in multi-injection insulin therapy in the remission group and one in metformin treatment in the discordant group). Both patients were taking insulin therapy at diagnosis: the first one maintained this therapy over time, whereas the second one gradually switched from insulin therapy to metformin.

Eight out of 11 patients affected by dyslipidemia took statin treatment; in particular, six took simvastatin (three in the remission group and three in the discordant group) and two rosuvastatin (one in the remission group and one in the discordant group). Six patients (33.3%) titered statin treatment since diagnosis (four in the remission group, two in the discordant group) (**Table 3**).

**Table 3:** Disease complications and associated comorbidities at the last follow-up.

Disease complications	Total (n = 18)	Group A (biochemical remission) (n = 9)	Group B (discordant GH/IGF-1) (n = 9)	p value
Hypertension, n (%) - 1 drug, n (%) - More than 1 drug, n (%) - Therapy potentiation, n (%)	11 (61.1) 4 (22.2) 7 (38.8) 5 (27.8)	5 (27.8) 2 (11.1) 3 (16.7) 2 (11.1)	6 (33.3) 2 (11.1) 4 (22.2) 3 (16.7)	NS
Diabetes mellitus, n (%) - Nutritional therapy, n (%) - Metformin, n (%) - Insulin, n (%)	6 (33.3) 4 (22.2) 1 (5.6) 1 (5.6)	3 (16.7) 2 (11.1) 0 1 (5.6)	3 (16.7) 2 (11.1) 1 (5.6) 0	NS
Dyslipidemia, n (%) - Statin treatment, n (%) - Simvastatin, n (%) - Rosuvastatin, n (%) - Therapy potentiation, n (%)	11 (61.1) 8 (44.4) 6 (33.3) 2 (11.1) 6 (33.3)	5 (27.8) 4 (22.2) 3 (16.7) 1 (5.6) 4 (22.2)	6 (33.3) 4 (22.2) 3 (16.7) 1 (5.6) 2 (11.1)	NS

NS, not significant.

Considering the number of drugs or the shift in medications, no statistically significant differences between groups were found (**Table 3**).

Focusing on the two patients in SSA only (group A), one of them was not affected by metabolic complications at diagnosis and during follow-up; the second patient was affected at diagnosis by arterial hypertension and dyslipidemia, needing the titration of treatment during follow-up, and new onset of diabetes mellitus in medical nutritional therapy was documented.

## Discussion

A proper phenotyping of acromegaly is crucial in order to diagnose and treat the pathology in early stages, when irreversible complications have not occurred yet. Organ-specific complications of acromegaly should improve or even be prevented by normalization of the GH and IGF-1 levels, and disease control could reduce the mortality risk as for the general population (20). The description of discrepancy between the principal parameters used to define acromegaly activity (namely, GH and IGF-1) is not a rare phenomenon; it represents a challenge since it can cause misunderstanding for the clinician leading to a complicated management and anxiety for the patient, and finally, it confuses the complex scenario of this systemic disease (21). The discrepancy should be referred to elevated GH levels with normal IGF-1 for sex and age (i.e., “High GH” discrepancy), and it usually implies a possible deregulation in GH pulsatility. Less frequently, “High IGF-1” discrepancy should exist, characterized by abnormal IGF-1 with normal GH levels, due to an enhanced responsiveness of peripheral tissues to circulating GH (21). In both cases, the clinical awareness of variables and conditions affecting GH and IGF-1 levels is important to interpret discordant results and to carry on a proper follow-up. To note, a recent Italian study demonstrated that the mean of three GH values collected during consecutive patients’ evaluations lessened the impact of GH cutoffs on discordance with IGF-1 (22). Similarly, Bona et al. (23) showed that the accuracy of the mean GH profile, associated with IGF-1, is higher than a single fasting GH measurement.

In the present study, we explored the impact of GH/IGF-1 discordance on the onset and aggressiveness of metabolic complications in acromegalic operated patients. Due to the rarity of the disease, usually most results derived from multicentric series, compromising the homogeneity of the population. The present research describes the clinical and biochemical characteristics of acromegaly in a group of homogeneous patients evaluated in a single center of tertiary care.

Previous studies investigated the impact of discrepancy on diabetes and arterial hypertension (16), whereas, to the best of our knowledge, it is the first study that has tried to associate hormone discrepancy also with dyslipidemia.

Considering diabetes and arterial hypertension, we did not find any difference between groups. The same results were obtained in the recent study by Amodru et al. on 190 patients recruited in eight European centers (16) that did not demonstrate any adverse outcome for hyperglycemia or arterial hypertension in acromegalic patients with discordant GH/IGF-1 values, either in High GH or in High IGF-1 patients. Other studies (24, 25) documented higher fasting glucose and glycated hemoglobin levels in the IGF-1 discordant group. The recent study by Campana et al. (22), who divided patients in controlled/high GH/high IGF-1/active disease, did not describe differences in the prevalence of comorbidities except for a trend of higher prevalence of diabetes in active disease and “high IGF-1” near to significance. The different results between studies should be explained with (i) the different criteria used for discrepancy, (ii) the evaluation of differences in terms of blood tests as glycemia and HbA1c (24), (iii) or the onset of diabetes or changing in antidiabetic drugs with more aggressiveness (16), as in our study.

Regarding arterial hypertension, the research by Matta et al. demonstrated that discordant patients had higher systolic blood pressure compared with the control group (130 vs. 120 mmHg) (25); again, the definition of discrepancy was different (patients with elevated IGF-1 and normal GH) than in our study (both High GH and High IGF-1 patients) and the evaluation of the *in office* blood pressure or the need to change medication could affect different results.

Furthermore, the impact of BMI on metabolic complications should be considered. In the study on the Liege Acromegaly Survey database (16), an increase of BMI was observed during follow-up, but without having an impact on the occurrence of metabolic comorbidities. The authors explained these data with two hypotheses: first, the higher BMI was related to aging; second, biochemical control of acromegaly is correlated with higher fat mass and total body weight (26). Furthermore, body composition could be another key of lecture, but no studies have considered this aspect yet.

The impact of GH/IGF-1 discrepancy on dyslipidemia has not been explored yet. The prevalence of dyslipidemia in acromegalic patients ranges from 13 to 51% according to the studies, and it is expected at diagnosis. In fact, GH causes lipolysis that results in FFA releasing into bloodstream, leading to an “inflammatory” microenvironment of the adipose tissue (11). Thus, a high level of triglycerides and a low level of HDL are the principal alterations of lipid metabolism in patients affected by acromegaly (5). IGF-I mediates GH actions, increases the anabolic actions of GH, and contrasts its detrimental effects (i.e., lipolysis, gluconeogenesis, and reduction of insulin action). Moreover, basic studies demonstrated the lowering of circulating plasma FFAs after high load of IGF-I infusion. Thus, considering that triglycerides stored in fat cells are the major pool of circulating FFAs, the reduction of plasma FFA could be a consequence of an inhibition of lipolysis (11). As for the other metabolic complication, we did not find any difference within groups.

Other studies showed a reduction of quality of life in acromegalic patients, without consistent differences in discordant than concordant groups, even though this was not evaluated in our cohort (27).

Regarding gender difference, our study underlined, as in other cohorts (16), a predominance of women in the whole population and in the remission group. The research by Alexoupoulou et al. (24), who divided patients in High IGF-1, High GH, remission, and active disease, showed a significant smaller prevalence of female subjects in the High IGF-1 group (36%) than in the High GH group (72%), hypothesizing a role for circulating estrogen in women as a cause for GH resistance (28), which finally showed a biochemical pattern of “high” GH and “low” IGF-1 (28).

Considering the whole population, IGF-I levels at last follow-up were positively correlated with IGF-1 at diagnosis and negatively correlated with age at diagnosis and length of SSA treatment. These results are not surprising since older patients compared with younger ones could present a milder phenotype, explained by different reasons as smaller and enclosed tumors and lower levels of GH and IGF-1 (29). In fact, the GH/IGF-I axis is characterized by a decrease of activity with aging. In adulthood, a decline of GH release is described and, in the elderly, a further reduction in daily GH secretion exists, due to a concomitant decrease in the GH peaks frequency and amplitude. The term *somatopause* has been suggested to describe the clinical modifications related to aging (i.e., sarcopenia, osteopenia, increased visceral adiposity, insulin resistance) possibly due to a decrease in GH concentration. The reduction of GH levels of elderly is maintained in the acromegalic subjects, and large literature demonstrated that post-load GH nadir negatively correlates with age (30).

In our population, IGF-1 at short-term follow-up was higher than IGF-1 at long-term follow-up. In fact, remission of acromegaly is usually assessed 3 months after neurosurgery, when IGF-1 levels stabilize, but long-term biochemical control could be reached several years after initial surgery (31). In literature, factors associated with low remission rate are cavernous sinus invasion, larger tumor size, and higher preoperative GH levels. Regarding IGF-1, the levels respond linearly to GH concentration only up to a definite level and then reach a plateau at higher GH concentrations, which may explain why IGF-1 concentrations at diagnosis are less predictive for remission (32). Thus, consensus on acromegaly management recommended to wait at least 12 weeks after surgery to assess IGF-1 levels, as the postoperative decline in IGF-1 levels can be delayed compared with that of GH levels (33).

Our study should be interpreted in the light of some limitations, firstly the small sample size, due to our choice to include patients of a single center only, to ensure homogeneity of population and treatments; despite the small number of patients included in the study, a standardized management of acromegaly, metabolic complications, and therapies is guaranteed. The small cohort did not allow us to analyze differences between the High IGF-1 or High GH subgroups. Furthermore, detailed body composition was not investigated and should be a challenge topic in further studies. Another limitation is the lack of information on menopausal state, since gonadal status could influence both metabolic complications and IGF-1 values.

In conclusion, our study underlines, with a real-life approach, that GH/IGF-1 incongruence does not seem to represent a higher risk of metabolic complications, even dyslipidemia, in acromegalic patients, with results aligned with other recent studies. Metabolic complications, mainly dyslipidemia, are frequent in cured acromegalic patients, but GH/IGF-1 discrepancy does not seem to represent a risk factor for their presence or persistence. Thus, in absence of other parameters suspected for an active disease, patients with discordant values do not need a closer follow-up to reduce the risk of cardiovascular complications that, finally, affect survival. These findings should support the proper phenotyping of acromegaly, characterized by a complex systemic scenario, and are crucial in order to help the clinicians to ensure the optimal delivery of care and management of the disease, avoiding overmedicalization, and relieving the anxiety of patients that lead, finally, to a better quality of life.

## Data availability statement

The raw data supporting the conclusions of this article will be made available by the authors, without undue reservation.

## Ethics statement

The studies involving humans were approved by AOU “Maggiore della Carità” Novara. The studies were conducted in accordance with the local legislation and institutional requirements. The participants provided their written informed consent to participate in this study.

## Author contributions

MR: Writing – original draft. RP: Writing – original draft. AF: Writing – original draft. FPi: Writing – original draft. SC: Writing – original draft. VA: Writing – original draft. PM: Writing – review & editing. GA: Writing – review & editing. FPr: Data curation, Funding acquisition, Methodology, Writing – review & editing. MC: Conceptualization, Data curation, Investigation, Methodology, Writing – original draft, Writing – review & editing.
